# IDH1^R132H^ is intrinsically tumor-suppressive but functionally attenuated by the glutamate-rich cerebral environment

**DOI:** 10.18632/oncotarget.26203

**Published:** 2018-10-12

**Authors:** Patricia D.B. Tiburcio, Bing Xiao, Yi Chai, Sydney Asper, Sheryl R. Tripp, David L. Gillespie, Randy L. Jensen, L. Eric Huang

**Affiliations:** ^1^ Department of Neurosurgery, Clinical Neurosciences Center, University of Utah, Salt Lake City, Utah, USA; ^2^ Department of Oncological Sciences, Huntsman Cancer Institute, University of Utah, Salt Lake City, Utah, USA; ^3^ Department of Neurosurgery, Nanchang University Second Affiliated Hospital, Nanchang, Jiangxi, People's Republic of China; ^4^ ARUP Institute for Clinical and Experimental Pathology, Salt Lake City, Utah, USA

**Keywords:** glioma, glutamate, isocitrate dehydrogenase 1, mouse model, RCAS

## Abstract

Recurrent heterozygous mutation of isocitrate dehydrogenase 1 gene (*IDH1*), predominantly resulting in histidine substitution at arginine 132, was first identified in glioma. The biological significance of IDH1^R132H^, however, has been controversial, and its prevalent association with glioma remains enigmatic. Although recent studies indicate that IDH1^R132H^ is nonessential to tumor growth or even anti-tumor growth, whether IDH1^R132H^ initiates gliomagenesis remains obscure. In this study, we report that IDH1^R132H^ is intrinsically tumor-suppressive but the activity can be attenuated by glutamate—the cerebral neurotransmitter. We observed that IDH1^R132H^ was highly suppressive of subcutaneous tumor growth driven by platelet-derived growth factor B (PDGFB), but IDH1^R132H^ tumor growth and glioma penetrance were virtually indistinguishable from those of IDH1-wildtype tumors in orthotopic models. *In vitro*, addition of glutamate compromised IDH1^R132H^ inhibition of neurosphere genesis, indicating glutamate promotion of oncogenic dominance. Furthermore, we observed that *IDH1^R132H^* expression was markedly decreased in tumors but became more permissible upon the deletion of tumor-suppressor gene *Cdkn2a*. To provide direct evidence for the opposing effect of IDH1^R132H^ on PDGFB-driven glioma development, we explored tandem expression of the two molecules from a single transcript to preclude selection against *IDH1^R132H^* expression. Our results demonstrate that when juxtaposed with oncogenic PDGFB, IDH1^R132H^ overrides the oncogenic activity and obliterates neurosphere genesis and gliomagenesis even in the glutamate-rich microenvironment. We propose therefore that IDH1^R132H^ is intrinsically suppressive of glioma initiation and growth but such tumor-suppressive activity is compromised by the glutamate-rich cerebral cortex, thereby offering a unifying hypothesis for the perplexing role of IDH1^R132H^ in glioma initiation and growth.

## INTRODUCTION

Heterozygous mutations in the isocitrate dehydrogenase 1 (*IDH1*) gene are found most frequently in glioma, predominantly resulting in the mutant enzyme IDH1^R132H^ with histidine substitution at arginine 132 [1–3]. The biological function of *IDH1^R132H^*, however, remains controversial. The prevailing belief is that IDH1^R132H^ is oncogenic owing to the gain of neomorphic activity that converts 2-oxoglutarate (aka α-ketoglutarate)—the product of wild-type IDH1—in an NADPH-dependent reduction to an “oncometabolite” D-2-hydroxyglutarate (D2-HG), which in turn inhibits a class of 2-oxoglutarate-dependent dioxygenases involved in epigenetic regulation, collagen synthesis, and cell signaling [4]. Supporting evidence for this theory includes 1) the association of *IDH1* mutations with glioma evolution, glioma CpG island methylator phenotype, and proneural subtype; 2) the induction of methylator phenotype in normal human astrocyte by *IDH1^R132H^* transduction or D2-HG treatment; and 3) the association of *IDH1* mutations with repressive histone methylation marks that contribute to a less differentiated or stem-like state [5]. Despite the circumstantial evidence, the exact mechanism by which *IDH1^R132H^* drives glioma initiation remains ill-defined, and, moreover, evidence from recent studies apparently challenges this belief.

Specifically, despite effective reduction of D2-HG by small-molecule inhibitors specific to mutant IDH1, treated glioma cells, unexpectedly, accelerated proliferation and shortened survival in an animal model [6]. Therapeutic sensitivity is important to improved survival of glioma patients with *IDH1* mutations, but mutant IDH1 inhibitors desensitized tumors cells to irradiation and chemotherapy [7]. Apparently, these counterintuitive findings not only argue against therapeutic targeting of *IDH1* mutations but also question the presumptive oncogenic activity of IDH1^R132H^. Consistently, previous studies showed that *IDH1^R132H^* transduction inhibited rather than stimulated tumor growth [8, 9], and gliomas with *IDH1* mutations possessed attenuated oncogenic signaling in comparison with those without [8, 10–13]. These studies have led us to posit that *IDH1* mutations are tumor-suppressive on the contrary; the biological consequence of *IDH1* mutations in glioma is to ameliorate patient survival, at least in part, by inhibiting oncogenic signaling [13]. This concept is in accordance with the experimental demonstration of anti-tumor effects of D2-HG, which decreases the stability of *MYC*/*CEBPA* transcripts via *N*^6^-methyladenosine RNA modification and thereby inhibits tumor cell survival and proliferation [14]. We have reported recently that whereas heterozygous but not hemizygous *IDH1^R132H^* suppresses anchorage-independent growth of glioma cells, the surviving cells conversely selects against *IDH1^R132H^* heterozygosity [15]. Our findings not only support the concept of *IDH1^R132H^* being anti-oncogenic but also suggest the strong antagonism between tumor growth and heterozygous *IDH1^R132H^* expression in the experimental setting. This interpretation is consistent with the requirement of a wild-type *IDH1* allele for D2-HG production [16, 17] and the frequent loss of either wild-type or mutant *IDH1* allele in patient-derived xenograft, *ex vivo* neurosphere culture, and glioma recurrence and progression [11, 16, 18, 19], even though the underlying mechanism of copy number alteration remains unclear.

The concept that *IDH1^R132H^* heterozygosity is anti-oncogenic and incompatible with tumor growth seems at odds with the fact that greater than 70% of WHO grade II and grade III gliomas and secondary glioblastomas harbor *IDH1* mutations [1–3]. Moreover, IDH1^R132H^-specific inhibitor and mutant IDH1 pan-inhibitor have been shown to be effective in animal studies [20, 21]. It is noteworthy, however, that the anti-oncogenic activity of heterozygous *IDH1^R132H^* can be circumvented by genetic and metabolic alterations, including the loss of *IDH1^R132H^* heterozygosity and use of reducing equivalent [15]. Furthermore, deletion or amplification of either mutant or wild-type *IDH1* allele decreases D2-HG in glioma recurrence [19]. Moreover, glutamate—a neurotransmitter rich in the cerebral cortex—is sufficient to bypass the inhibitory effect of IDH1^R132H^ on glioma progenitor proliferation [9]. These findings altogether indicate the delicate nature of heterozygous *IDH1^R132H^*, whose tumor-suppressive activity can be compromised by genetic alterations and tumor microenvironment.

## RESULTS

### IDH1^R132H^ transduction suppresses subcutaneous tumor growth

We reported recently that heterozygous *IDH1^R132H^* is functionally anti-oncogenic, as evidenced by the antagonism between *IDH1^R132H^* heterozygosity and anchorage-independent growth; whereas heterozygous *IDH1^R132H^* suppressed neurosphere genesis, the surviving neurosphere selected against the expression of either *IDH1^R132H^* or *IDH1* transgene and reduced D2-HG levels [15]. To ascertain the tumor-suppressive activity of *IDH1^R132H^ in vivo*, we first established subcutaneous tumor growth of mouse astrocyte NA1 that had been transduced with luc–PDGFB, which expresses luciferase and platelet-derived growth factor B (PDGFB) upon P2A cleavage [15]. PDGFB has been used extensively for gliomagenesis *in vivo* [9, 22–26]. Accordingly, the transduced NA1 developed robust tumor growth with a volume-based doubling time of 9.4 days in contrast to NA1 transduced with luc*, which expresses the same transcript harboring a stop codon engineered at the P2A (Supplementary Figure 1).

**Figure 1:**
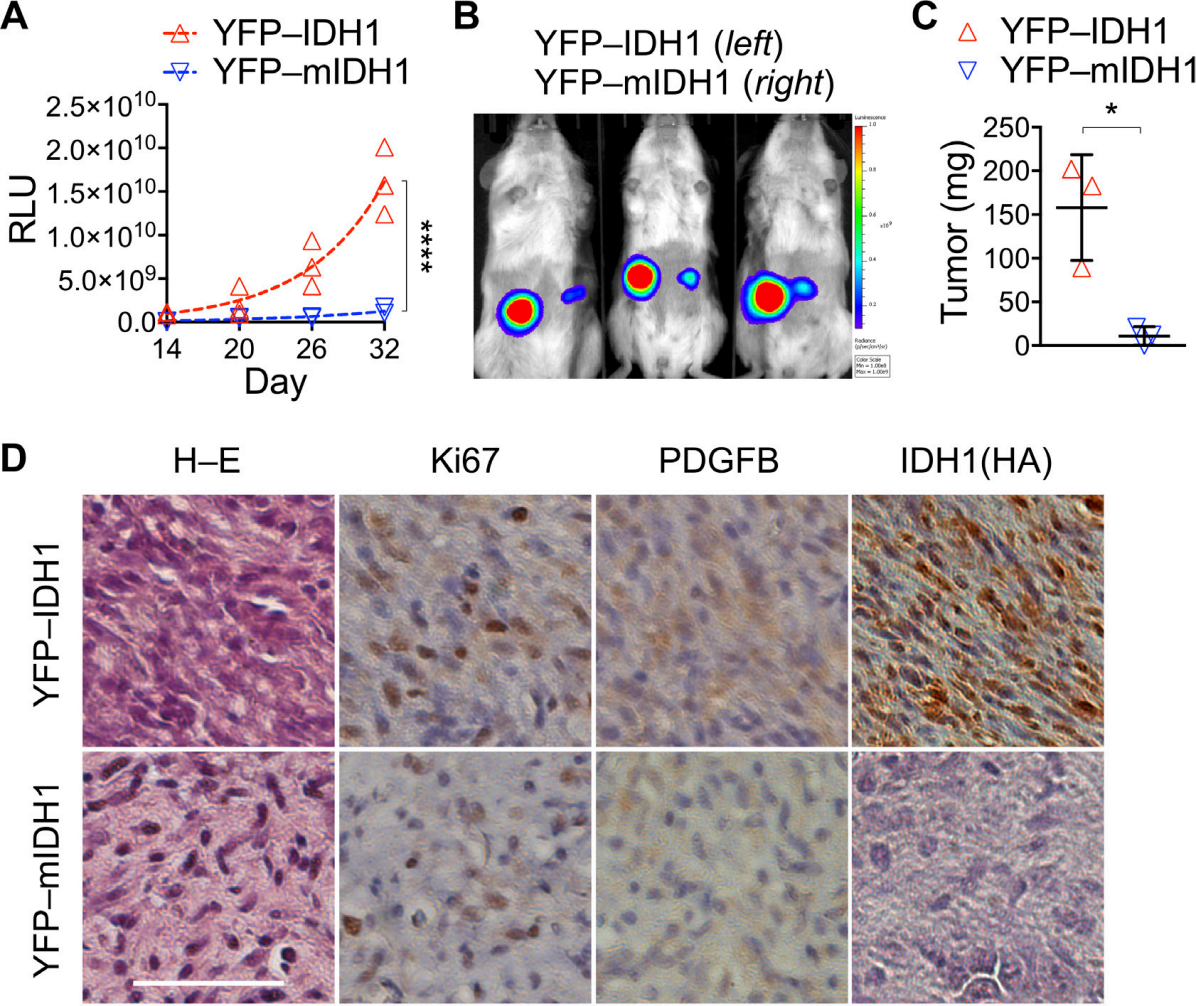
*IDH1^R132H^* transduction suppresses subcutaneous tumor growth (**A–C**) Subcutaneous tumors derived from NA1 astrocytes that had been transduced with luc–PDGFB showing significant growth suppression by co-transduction with YFP–IDH1^R132H^ (YFP-mIDH1) compared with YFP–IDH1 (**A**). Nonlinear regression curve fit was performed using exponential growth equation and two-way ANOVA for the analysis of statistical significance. RLU, relative luminescent units. ^****^p < 0.0001. Suppression of tumor growth was supported by bioluminescent imaging (**B**) and autopsied tumor weight (**C**). Unpaired *t*-tests were performed using two-tailed *p* value. ^*^p < 0.05. (**D**) Hematoxylin–eosin (H–E) and immunohistochemistry staining revealed less malignant histologic features, decreased Ki67 and PDGFB expression, and weak HA-tagged IDH1^R132H^ staining in YFP–IDH1^R132H^ tumor compared with YFP–IDH1 tumor. Scale bar: 50 μm.

Next, we sought to test whether *IDH1^R132H^* co-transduction inhibits tumor growth using YFP–IDH1^R132H^, which expresses nuclear yellow fluorescent protein (YFP) and IDH1^R132H^ upon P2A cleavage [15]. As expected, YFP–IDH1^R132H^ significantly inhibited cell proliferation, resulting in 20% increase of the mean doubling time to 28.8 hours compared with 24.0 hours of its control YFP–IDH1 (Supplementary Figure 2A). In keeping with this, YFP–IDH1^R132H^ cells showed G_2_/M arrest compared with YFP–IDH1 cells (Supplementary Figure 2B). In agreement with its effect on neurosphere genesis [15], YFP–IDH1^R132H^ markedly inhibited tumor growth, as indicated by bioluminescent imaging and confirmed by tumor weight analysis (Figure 1A–1C). The mean volume-based doubling time of YFP–IDH1^R132H^ tumors increased 66% to 15.8 days from 9.5 for YFP–IDH1 tumors. Histological examination confirmed reduced cellularity, nuclear–cytoplasm ratio, and nuclear pleomorphism in YFP–IDH1^R132H^ tumors compared with YFP–IDH1 tumors (Figure 1D). Furthermore, immunohistochemistry showed decreased Ki67 as well as PDGFB staining in YFP–IDH1^R132H^ tumors. It is noteworthy, however, in contrast to the staining of HA-tagged wild-type IDH1 in the control, HA-tagged IDH1^R132H^ was nearly undetectable in YFP–IDH1^R132H^ tumors and sparsely stained with an anti-IDH1^R132H^ antibody (Supplementary Figure 2C). Taken together, these results support the concept that IDH1^R132H^ is not only tumor-suppressive but is also selected against in the surviving tumors, as reported previously in anchorage-independent growth [15].

### Antagonism between *IDH1^R132H^* transgene expression and tumor growth

**Figure 2:**
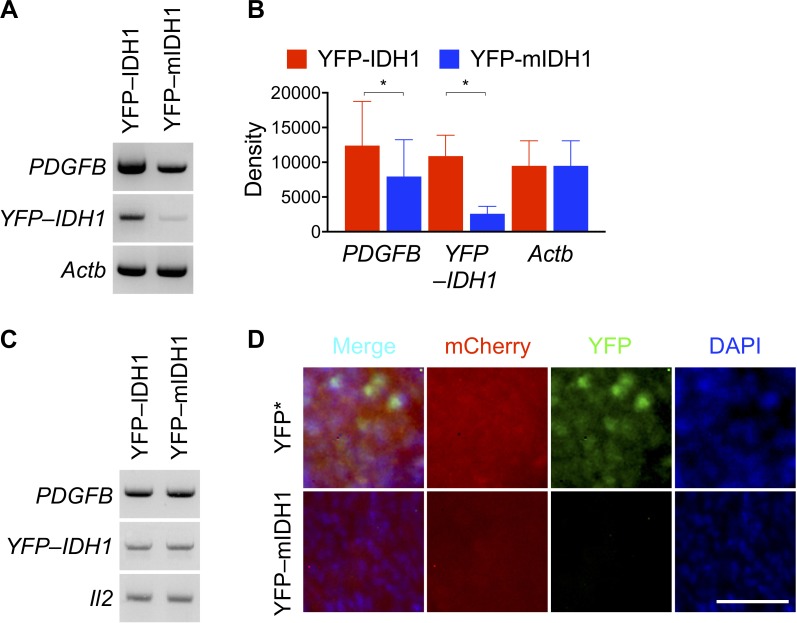
Markedly decreased YFP–IDH1^R132H^ expression in subcutaneous tumor (**A–C**) Reverse transcription–PCR showing marked decrease of *YFP*–*IDH1^R132H^* mRNA levels in the subcutaneous tumor along with modest decrease of *PDGFB* mRNA levels (**A**). Densitometry analysis supporting significant decreases of YFP–IDH1^R132H^ (*n* = 3) and PDGFB (*n* = 5) mRNA levels (**B**). PCR amplification of genomic DNA showing no copy number alterations (**C**). (**D**) Fluorescent microscopy confirming loss of IDH1^R132H^ expression and decreased PDGFB expression, as indicated by respective YFP and mCherry signals, in subcutaneous tumor derived from NA1 transduced with mCherry–PDGFB. Scale bar: 50 μm.

To provide further evidence for the selection against *IDH1^R132H^* expression in tumor growth, we observed 76% reduction of YFP–IDH1^R132H^ transcript levels accompanied by 35% reduction of *PDGFB* transcript levels compared with those in YFP–IDH1 tumors (Figure 2A, 2B), a finding in agreement with the selection against *IDH1^R132H^* transgene in neurosphere culture [15]. No reduction of *IDH1^R132H^* copy number, however, was observed at the genomic DNA level (Figure 2C), suggesting non-genetic event(s) for *IDH1^R132H^* downregulation.

Next, we employed fluorescent microscopy to visualize the suppression of *IDH1^R132H^* transgene expression by examining tumor cells transduced with mCherry–PDGFB, which expresses the fluorescent mCherry and PDGFB upon P2A cleavage [15]. To that end, we transduced NA1 cells with mCherry–PDGFB and YFP–IDH1^R132H^ or its control YFP*, which expresses only YFP protein from the same YFP–IDH1^R132H^ transcript that harbors an engineered stop codon [15]. Of note, we opted for YFP* as a more appropriate control because the upregulation of wild-type *IDH1* promotes aggressive growth of malignant glioma [27]. As expected, YFP–IDH1^R132H^ co-transduction resulted in significant decreases in cell proliferation and subcutaneous tumor growth compared with YFP* co-transduction (Supplementary Figure 3). Fluorescent microscopy revealed few cells that were YFP^+^ in the surviving YFP–IDH1^R132H^ tumor in contrast to widespread YFP^+^ cells in the control (Figure 2D), which is consistent with the previous finding in neurosphere culture [15]. We conclude therefore that although *IDH1^R132H^* transduction suppresses subcutaneous tumor growth, the surviving tumors, conversely, select against the transgene expression.

### IDH1^R132H^ tumors are histologically indistinguishable from IDH1-wildtype tumors in orthotopic models

**Figure 3:**
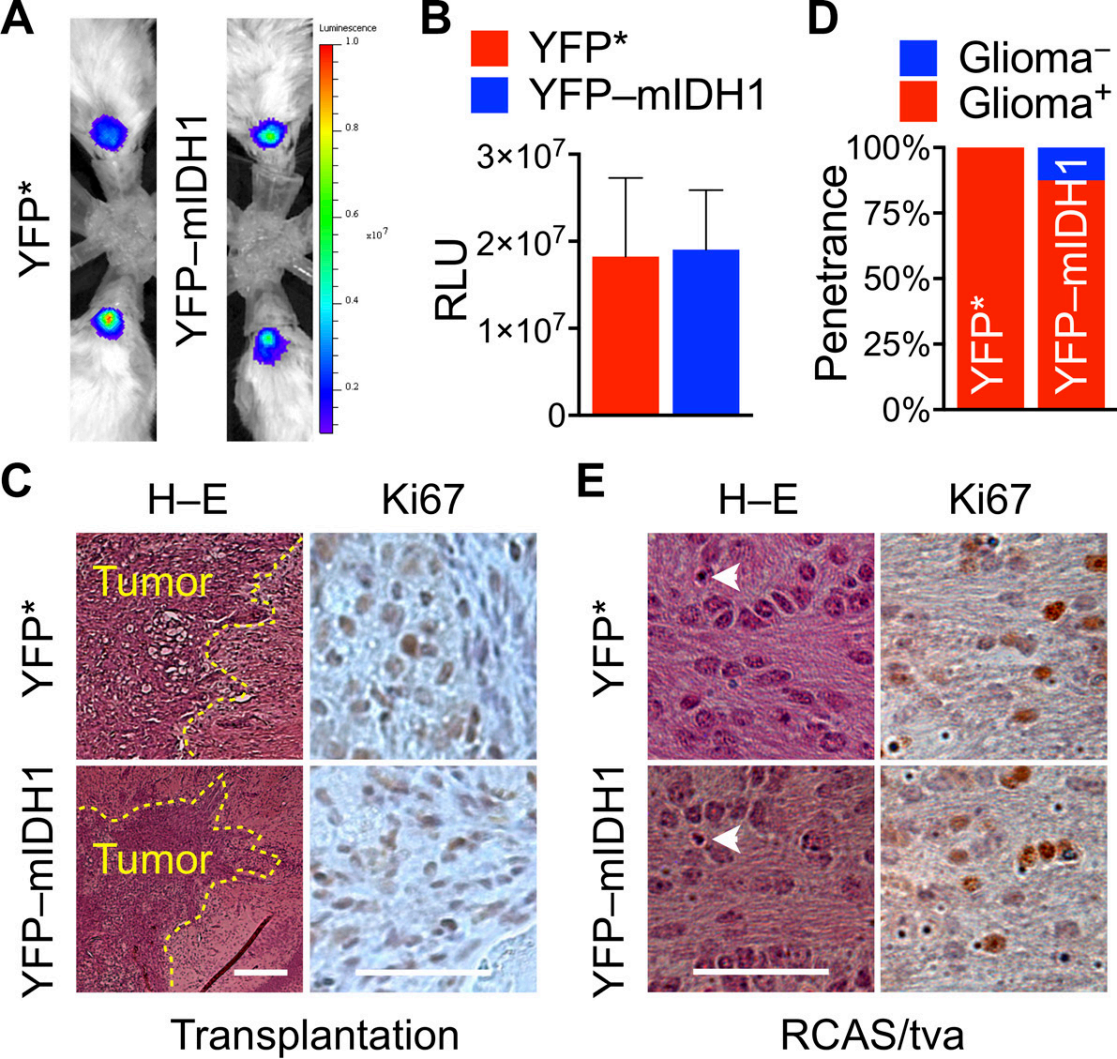
Loss of IDH1^R132H^ suppression of tumor initiation and growth in orthotopic models (**A–C**) YFP–IDH1^R132H^ failed to suppress PDGFB-driven tumor growth in intracranial transplantation compared with YFP*, as indicated by bioluminescent imaging (**A**), the mean bioluminescent values (**B**, *n* = 4), and invasive histologic presentation and similar patterns of Ki67 staining (**C**). RCAS/tva mouse model showing similar glioma penetrance (**D**) and malignant features including mitotic indices (arrowhead), infiltration, and Ki67 staining (**E**) between mice subjected to RCAS/PDGFB and RCAS/YFP* infection and RCAS/PDGFB and RCAS/YFP–IDH1^R132H^ infection (*n* = 8 for each group).

Unexpectedly, IDH1^R132H^ suppression of tumor growth could not be reproduced in orthotopic transplantation models, as shown by bioluminescent imaging and histological examination (Figure 3A, 3B). Indeed, YFP–IDH1^R132H^ transduction failed to inhibit PDGFB-driven orthotopic tumor growth, resulting in similar bioluminescent readings in reference to the control. Histological examination showed similar tumor cell proliferation and invasion in both groups of mice (Figure 3C), an observation consistent with previous reports [25, 28, 29]. The lack of clear tumor suppression in the orthotopic model indicates a tissue-specific role of the cerebral cortex in the biological effect of IDH1^R132H^.

We next sought to corroborate the tissue-specific effect of IDH1^R132H^ in a spontaneous glioma mouse model, which combines the replication-competent avian sarcoma-leukosis virus long-terminal repeat with splice acceptor (RCAS) for transgene delivery [30–32] with a transgenic line (*Nes*-*tva*) carrying Nestin promoter-driven expression of avian retroviral receptor tva [33]. PDGFB has been used as a potent inducer of gliomagenesis in *Nes*-*tva* mice [22, 24, 26, 34, 35]. Consistently, we observed equivalent glioma penetrance between mice co-transduced with PDGFB and YFP* (100% or 8/8) and those co-transduced with PDGFB and YFP–IDH1^R132H^ (88% or 7/8) (Figure 3D). Our earlier investigation using PDGFB and IDH1 or IDH1^R132H^ showed similar penetrance: 77% (7/9) or 75% (6/8). Furthermore, similar invasion of the cerebral cortex and Ki67 and Olig2 staining were observed in both tumor types (Figure 3E; Supplementary Figure 4), as shown previously [28, 29]. Taken together, these results suggest that the tumor-suppressive effect of IDH1^R132H^ is functionally compromised by the cerebral cortex in the experimental setting.

### IDH1^R132H^ expression becomes permissible in glioma with *Cdkn2a* deletion

**Figure 4:**
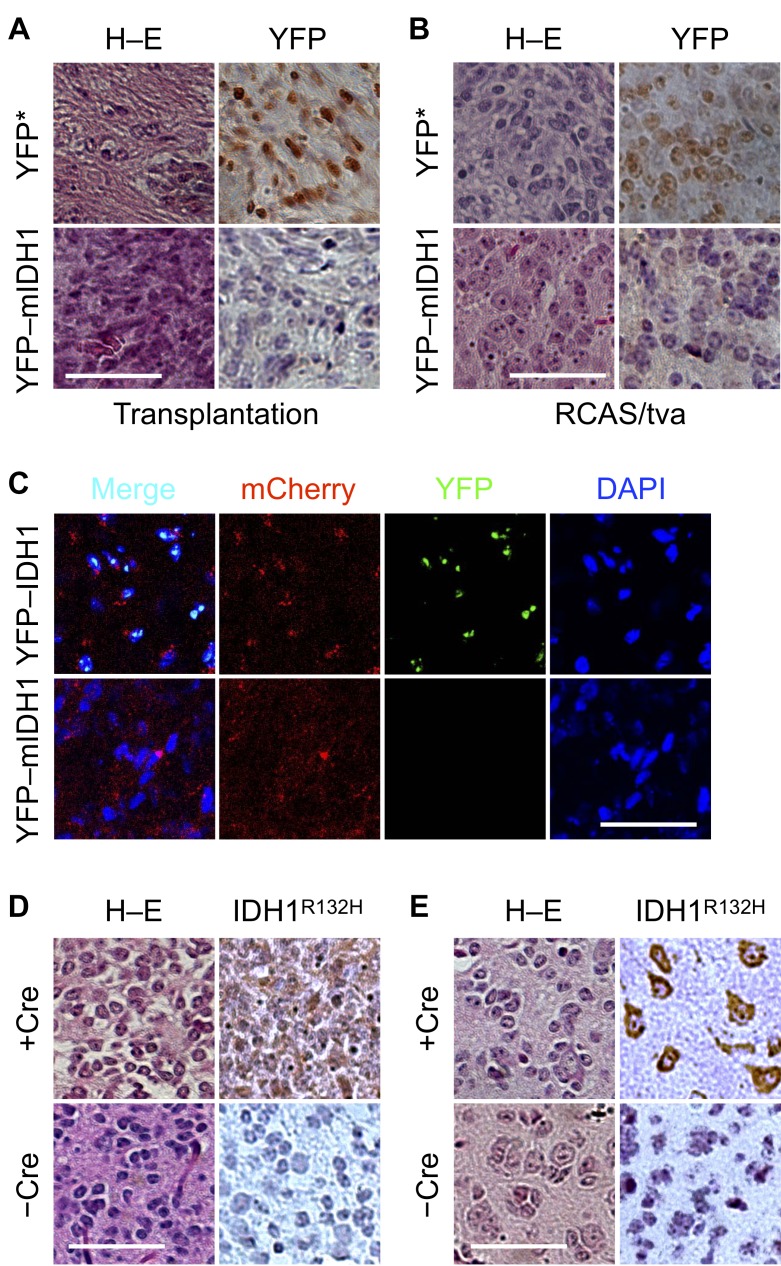
Dependence of IDH1^R132H^ expression on *Cdkn2a* deletion in orthotopic tumor Pronounced reduction of YFP staining in YFP–IDH1^R132H^ intracranial transplantation (**A**) and RCAS/tva glioma (**B**) models compared with their YFP* controls. (**C**) Fluorescent microscopy confirming the loss of YFP–IDH1^R132H^, but not YFP–IDH1, expression in RCAS/tva glioma driven by mCherry–PDGFB. *Cdkn2a* deletion with RCAS/Cre (+Cre) in *Nes-tva*;*Cdkn2a^fl/fl^* mice increased IDH1^R132H^ expression in the cytoplasm of tumor cells (**D**) and those involved in perineuronal satellitosis (**E**). Scale bar: 50 μm.

Although orthotopic transplantation exhibited similar tumor growth between YFP–IDH1^R132H^ and YFP* control cells, strong nuclear staining of YFP was seen in YFP*, but not YFP–IDH1^R132H^, tumors by immunohistochemistry (Figure 4A). Additionally, IDH1^R132H^ staining was scattered in YFP–IDH1^R132H^ tumor cells (Supplementary Figure 5A). Likewise, in the RCAS/PDGFB glioma model, weak YFP staining was seen in YFP–IDH1^R132H^ glioma cells in contrast to prevalent nuclear staining in YFP* glioma cells (Figure 4B; Supplementary Figure 5B). Furthermore, in RCAS/mCherry–PDGFB-induced gliomas, fluorescent YFP signal was visualized only in the YFP–IDH1, but not YFP–IDH1^R132H^, tumors despite a modest decrease of mCherry signal in the latter (Figure 4C). These results strongly indicate that *IDH1^R132H^* transgene expression is selected against during glial tumor growth irrespective of tumor size and microenvironment, supporting the notion of antagonism between *IDH1^R132H^* expression and tumor growth.

Our findings are apparently at odds with the fact that IDH1^R132H^ is detectable immunologically in human gliomas and tumor transplantations [10, 36–40]. In fact, IDH1^R132H^ staining was strong and conspicuous in RCAS/PDGFA gliomas when combined with *Cdkn2a* knockout and *Trp53* knockdown [28]. In light of frequent mutations in various tumor-suppressor genes associated with IDH-mutant glioma [41], we hypothesized that the inactivation of tumor-suppressor gene(s) renders glioma more permissible to *IDH1^R132H^* expression. To test this notion, we compared immunohistochemical IDH1^R132H^ staining between tumors developed in *Cdkn2a*-intact and -deleted genetic background. Indeed, IDH1^R132H^ gliomas derived from *Cdkn2a^fl/fl^* mice showed much increased immunohistochemical staining of IDH1^R132H^ with Cre co-transduction compared with those without (Figure 4D). Additionally, IDH1^R132H^ staining was seen in glioma cells forming perineuronal satellitosis (Figure 4E), as reported previously [36]. Taken together, these results support the selection against *IDH1^R132H^* transgene in PDGFB-driven tumors and the dependence of *IDH1^R132H^* expression on inactivation of tumor-suppressor gene(s).

### Expression of IDH1^R132H^ and PDGFB from the same transcript obliterates gliomagenesis

Although recent studies indicated anti-tumor effects of *IDH1^R132H^* [14, 19], whether *IDH1^R132H^* suppresses gliomagenesis remains unclear. To provide evidence that IDH1^R132H^ is tumor-suppressive, we engineered a RCAS vector that expresses IDH1^R132H^, P2A, and PDGFB from the same transcript. This tandem design of IDH1^R132H^–PDGFB not only ensures the expression of the two at 1:1 molar ratio within the same cells but also precludes selection against *IDH1^R132H^* expression especially in the cerebral cortex (Figure 4). Similarly, IDH1–PDGFB was constructed as control.

**Figure 5:**
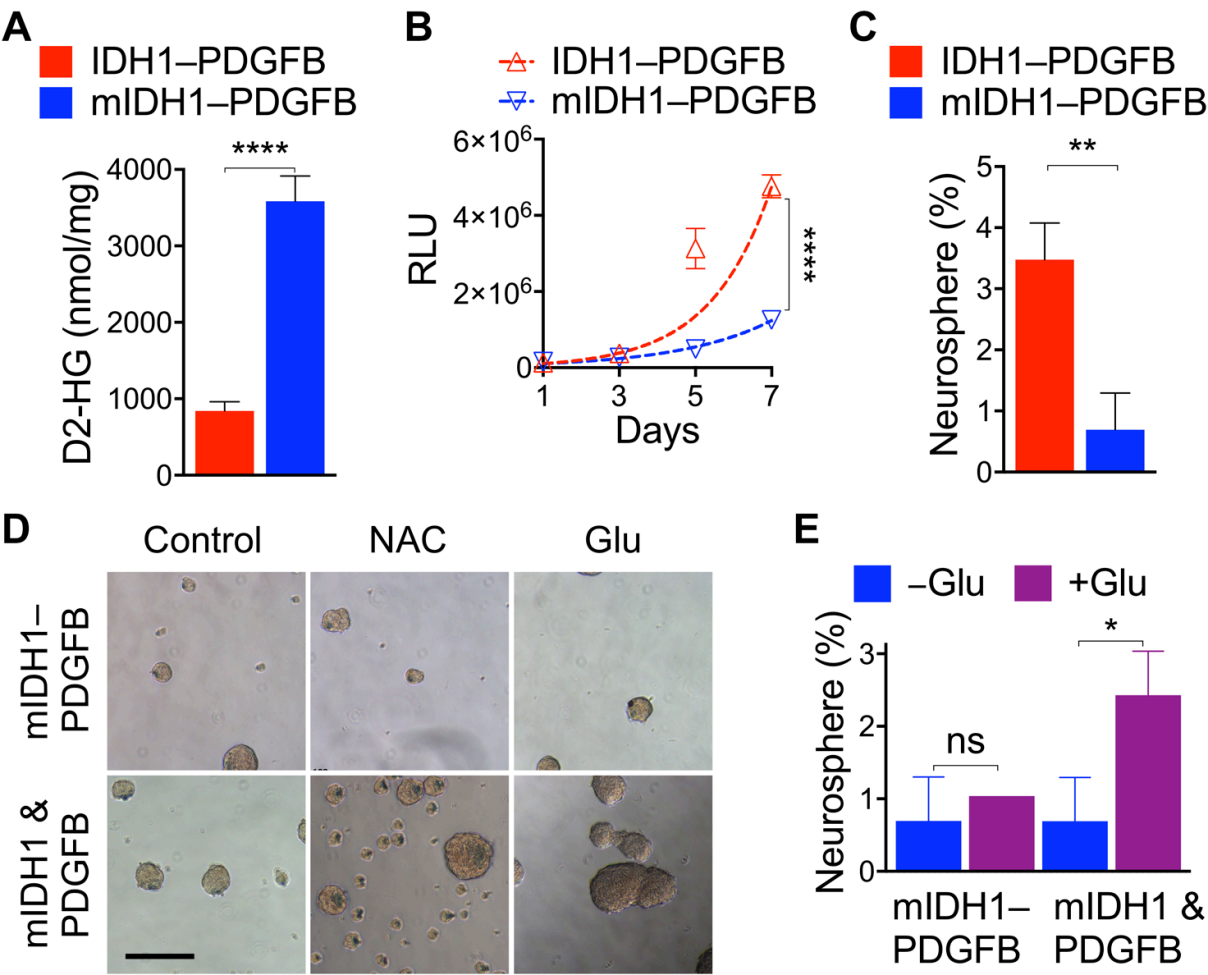
IDH1^R132H^ overrides oncogenic PDGFB in tandem expression (**A**) An extremely significant increase of D2-HG levels (*n* = 6) in NA1 transduced with IDH1^R132H^–PDGFB (mIDH1–PDGFB) in reference to the control. Significant decreases in NA1 proliferation (**B**, *n* = 6) and neurosphere genesis from single cells (**C**, *n* = 3) upon transduction with IDH1^R132H^–PDGFB compared with IDH1–PDGFB. (**D**) Treatment with 1 mM *N*-acetyl cysteine (NAC) or glutamate (Glu) failed to increase the size and number of neurosphere growth that had been transduced with IDH1^R132H^–PDGFB but did so in those co-transduced with YFP–IDH1^R132H^ and mCherry–PDGFB (mIDH1 & PDGFB). (**E**) Quantitative analysis confirming the ineffectiveness of glutamate on neurosphere genesis from single mIDH1–PDGFB cells (*n* = 3). Unpaired *t*-tests were performed using two-tailed *p* value. ns, not significant, ^*^p < 0.05; ^**^p < 0.01; ^****^p < 0.001. Scale bar: 200 μm.

We confirmed equivalent transgene expression between NA1 cells transduced with IDH1–PDGFB and IDH1^R132H^–PDGFB at mRNA and protein levels (Supplementary Figure 6A, 6B). We observed the mean D2-HG levels at 3,583 nmol per mg protein in IDH1^R132H^–PDGFB cells (Figure 5A), a concentration similar to those in human IDH1^R132H^ glioma cells [15] and fourfold greater than that in IDH1–PDGFB cells. IDH1^R132H^–PDGFB cells showed a remarkable decrease in proliferation compared with IDH1–PDGFB cells, resulting in 47% increase of doubling time to 41.9 hours from 28.5 (Figure 5B). Furthermore, we determined the ability of single cells to form neurosphere; consistent with the inhibitory effect of YFP–IDH1^R132H^ when co-expressed with luc–PDGFB or mCherry–PDGFB from different transcripts [15], IDH1^R132H^–PDGFB cells had a fivefold reduction of neurosphere genesis from 3.5% in IDH1–PDGFB cells to 0.7, a level equivalent to the parental NA1 (Figure 5C; Supplementary Figure 6C). These results indicate that IDH1^R132H^–PDGFB is a functional platform demonstrating that a single-nucleotide difference in *IDH1* is sufficient to confer suppression of anchorage-independent growth by overriding the oncogenic activity of PDGFB.

Previous studies have indicated the importance of glutamate anaplerosis in IDH-mutant glioma metabolism and growth [9, 42]. In particular, the addition of glutamate reversed IDH1^R132H^-mediated proliferative inhibition of neural progenitor cells co-transduced with PDGFB [9]. Likewise, we previously showed that the addition of reducing equivalent *N*-acetyl cysteine reversed inhibition of anchorage-independent growth by heterozygous *IDH1^R132H^* [15]. We sought to determine whether IDH1^R132H^–PDGFB cells would respond differently to the treatment of glutamate or *N*-acetyl cysteine in neurosphere genesis. Indeed, treatment with sodium glutamate or *N*-acetyl cysteine markedly increased size and number of PDGFB-driven neurospheres when YFP–IDH1^R132H^ was expressed from different transcripts (Figure 5D, *bottom*); however, neither treatment had noticeable effect on those transduced with IDH1^R132H^–PDGFB (*top*). Furthermore, results from single-cell analysis confirmed that glutamate treatment had virtually no effect on IDH1^R132H^–PDGFB cells in contrast to a 3.5-fold increase in the co-transduced cells from 0.69% to 2.43 (Figure 5E). Therefore, these results not only further support the concept that IDH1^R132H^ is intrinsically tumor-suppressive but also suggest a complete suppression of glioma development if IDH1^R132H^ is co-expressed with PDGFB from the same transcript.

**Figure 6:**
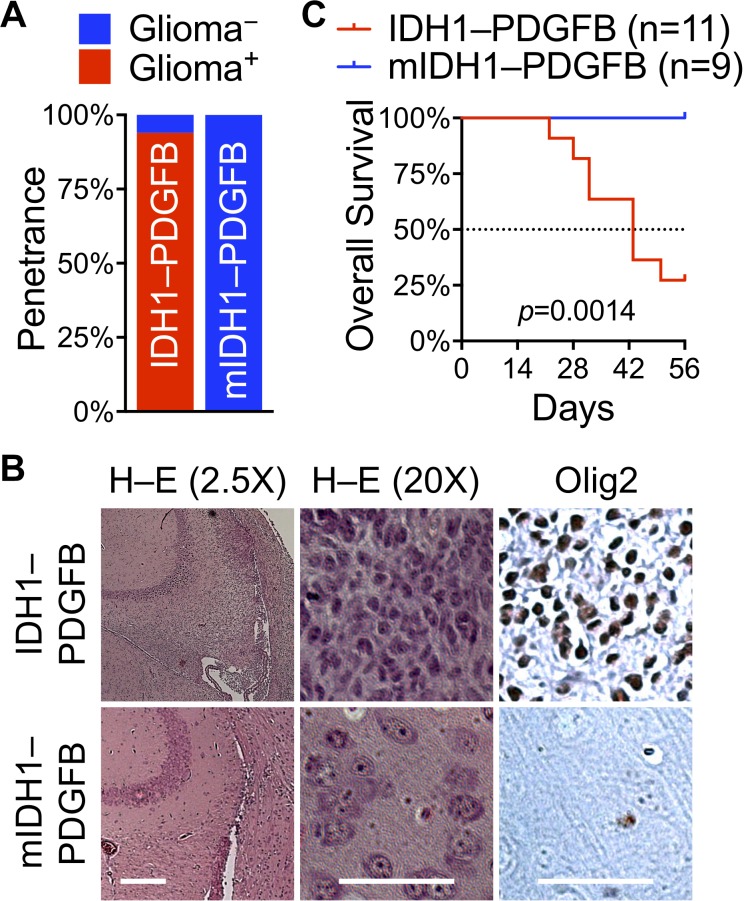
IDH1^R132H^ obliterates PDGFB-driven gliomagenesis when expressed in tandem (**A**) Ninety-three percent of glioma penetrance (Glioma^+^) with RCAS/IDH1–PDGFB (14/15) in contrast to zero percent of glioma penetrance (Glioma^−^) with RCAS/IDH1^R132H^–PDGFB (13/13) in *Nes-tva* mice. (**B**) Lack of glioma lesions in the brain of RCAS/IDH1^R132H^–PDGFB mice, as determined by histologic examination and Olig2 staining. Scale bar: short, 500 μm; long, 50 μm. (**C**) A Kaplan–Meier plot showing a striking survival difference between RCAS/IDH1–PDGFB mice and RCAS/IDH1^R132H^–PDGFB mice.

Given the overriding role of IDH1^R132H^ against oncogenic PDGFB in anchorage-independent growth, we predicted that expression of *IDH1^R132H^* with *PDGFB* from the same transcript would prevent spontaneous glioma initiation and growth even in the glutamine-rich microenvironment. Indeed, none of the mice (13/13) developed glioma with RCAS/IDH1^R132H^–PDGFB in contrast to 93% incidence (14/15) in those with RCAS/IDH1–PDGFB (Figure 6A, 6B). In addition, immunohistochemistry showed widespread Olig2 staining in tumor cells but not in the cortex of IDH1^R132H^–PDGFB mice (Figure 6B). Furthermore, Kaplan–Meier analysis revealed that IDH1^R132H^ was remarkably beneficial to the survival of IDH1^R132H^–PDGFB mice; none of them exhibited clear neurologic signs by the end of the 8-week period, whereas 73% IDH1–PDGFB mice had to be sacrificed, thereby significantly decreasing the median survival to 43 days (Figure 6C). Histological examination and Olig2 immunohistochemical staining confirmed the presence or absence of glioma lesions in all of the mice (data not shown). Thus, we conclude that IDH1^R132H^ is intrinsically suppressive of glioma initiation as well as glioma growth.

## DISCUSSION

We present evidence in this study that the outcome of *IDH1^R132H^* transduction in glioma initiation and growth is context dependent even though *IDH1^R132H^* is intrinsically tumor-suppressive. Specifically, we demonstrate that when *IDH1* and *PDGFB* are expressed from the same transcript, a single-nucleotide change of *IDH1* at codon 132 determines the fate of gliomagenesis and overall survival of *Nes-tva* mice. Our results provide direct evidence that IDH1^R132H^ is not only intrinsically tumor-suppressive but also resistant to functional compromise by the environmental glutamate or reducing power, which would otherwise attenuate the antagonism of IDH1^R132H^ toward the oncogenic PDGFB when both are expressed from different transcripts. This finding offers an explanation for the distinct effects of IDH1^R132H^ on tumor growth between the subcutaneous and the glutamate-rich cerebral environment in this study. Our observation that the addition of glutamate markedly decreased the inhibitory effect of *IDH1^R132H^* on neurosphere growth and genesis from single cells is in agreement with glutamate reversal of IDH1^R132H^ inhibition of neural progenitor cell proliferation [9] and is consistent with the metabolic dependence of IDH1-mutant glioma on glutamate [43]. Although we did not provide evidence that there is sufficient glutamate in the cerebral cortex to feed glioma in our models, these results nevertheless support the concept that IDH1^R132H^-mediated tumor suppression can be compromised by the microenvironmental factors including glutamate and reducing equivalent as escape mechanisms of glioma progression [15]. The study may also account for the prevalence of IDH mutations in glioma and the nonexistence in most other cancer types [2, 3, 44, 45]. Furthermore, our results may provide a clue to the challenging issue of maintaining *IDH1^R132H^* heterozygosity in glioma cell culture [11, 18, 46].

In agreement with our initial concept that the biological consequence of IDH mutations is antagonistic toward oncogenic signaling [13], the conclusion of *IDH1^R132H^* being intrinsically tumor-suppressive is further supported by the observations that IDH1^R132H^ is anti-tumor growth or incompatible with glioma progression [6, 8–10, 14, 15, 19, 28]. Moreover, genetic studies indicate that endogenous *Idh1^R132H/+^* expression alone is non-tumorigenic in hematopoietic and neural tissues [29, 47–49]. Conditional expression of transgenic *IDH2* mutation in knock-in mice caused cardiomyopathy and neurodegeneration instead [50]. Importantly, endogenous *Idh1^R132H/+^* expression through *Nes*-*CreER^T2^* resulted in 70% decrease in glioma penetrance induced by *Trp53* deletion and extended mouse survival in reference to *Idh1* wild-type expression [29]. Likewise, *IDH1^R132H^* gliomas driven by *PDGFA* transduction and *Trp53* knockdown show significantly extended survival in comparison with *IDH1* gliomas [28]. Therefore, the genetic evidence supports the conclusion that *IDH1^R132H^* is intrinsically tumor-suppressive.

In accordance with the incompatibility between *IDH1^R132H^* heterozygosity and anchorage-independent growth [15], we observed the strong antagonism between IDH1^R132H^ expression and PDGFB-driven tumor growth. Interestingly, *IDH1^R132H^* transgene expression was markedly attenuated but more permissible at the expense of *Cdkn2a* deletion. We anticipate additional *Trp53* knockdown would result in even greater *IDH1^R132H^* transgene expression, as shown previously [28]. The biological consequence of tumor-suppressor gene inactivation, however, is the erosion of *IDH1^R132H^* tumor-suppressive activity, as indicated by the complete loss of *IDH1^R132H^* survival benefit in *Cdkn2a^−/−^* mice in contrast to *Cdkn2a^+/+^* and *Cdkn2a^−/+^* mice [28]. In light of the association of IDH-mutant gliomas with mutations in tumor-suppressor genes including *TP53*, *ATRX*, *CIC*, *NOTCH1*, and *FUBP1* [41], it stands to reason that IDH1^R132H^ expression becomes permissible and therefore detectable in these gliomas of various grades [36]. The notion that inactivation of tumor-suppressor gene(s) permits *IDH1^R132H^* existence and expression in glioma may account for the continuous presence of *IDH1^R132H^* in recurrent gliomas [51, 52], even though some recurrent gliomas underwent genetic deletion of mutant *IDH1* allele and copy number alterations [16, 19]. Whether *IDH1^R132H^* detected in recurrent glioma is fully functional remains to be investigated; the finding that *IDH1^R132H^* and D2-HG are nonessential at recurrence nevertheless has raised the question of targeting IDH1^R132H^ for therapeutic intervention [19]. It is interesting to note that although the IDH1^R132H^-specific inhibitor AGI-5198 was shown initially to be effective in inhibiting glioma cell growth in subcutaneous xenograft [20], followup studies found lack of tumor regression in the same tumor model [53]. Despite the high potency in 2HG suppression among available mutant IDH1 inhibitors, their therapeutic efficacies in survival experiments vary from modest to harmful [6, 21]. Additionally, studies of gliomagenesis in cell culture models also indicate that 2-HG is nonessential to cell growth [54].

Given the association of PDGFRα with IDH-mutant glioma [39, 55, 56], the use of *PDGFA* as an oncogenic driver seems more relevant because PDGFA activates only PDGFRα [57]. Furthermore, since *Trp53* deletion is sufficient to induce glioma [29], it will be interesting to test further whether *IDH1^R132H^* obliterates gliomagenesis driven by *PDGFA* transduction or *Trp53* knockdown when expressed from the same transcript. It should be noted that this design, albeit artificial, has enabled us to tease out the intrinsic function of *IDH1^R132H^*, similar to what the genetic engineering of heterozygous *IDH1^R132H/+^* in HCT116 colon cancer cells, for instance, has done to advance the understanding of glioma biology. Thus far, our tandem design arguably has the advantage of directly determining the antagonism between *IDH1^R132H^* and oncogenic activities in gliomagenesis.

In summary, this study has shown that *IDH1^R132H^* is intrinsically tumor-suppressive, and yet its anti-tumor activity can be compromised by internal factors, such as inactivation of tumor-suppressor gene, and external factors, such as glutamate. The context-dependent effects of *IDH1^R132H^* on tumor initiation and growth may have implications in glioma etiology, model development, and therapeutic targeting.

## MATERIALS AND METHODS

### RCAS vectors and retroviral generation

All RCAS vectors including RCAS/luc–PDGFB, RCAS/mCherry–PDGFB, RCAS/YFP–IDH1, RCAS/YFP–IDH1^R132H^, and RCAS/YFP* were created as described previously [15]. RCAS/IDH1–PDGFB and RCAS/IDH1^R132H^–PDGFB vectors were constructed using the Gibson assembly (New England Biolabs) as described previously [15]. RCAS/luc* was derived from RCAS/luc–PDGFB with the introduction of a stop codon at P2A. Recombinant retroviruses were generated essentially as described previously [15, 26].

### Cell culture, retroviral infection, and neurosphere culture

Immortalized mouse astrocyte cell line NA1 was prepared and subjected to retroviral infection as described previously [15]. Likewise, resultant cells with fluorescent signals were enriched by flow cytometry, and the IDH1^R132H^ status was verified by DNA sequencing. Neurosphere growth was compared qualitatively by seeding 10,000 cells in a 48-well plate with 500 μL of neural stem cell medium [Neurobasal media supplemented with B-27, 10 ng/mL bFGF, and 20 ng/mL EGF (Invitrogen)]. An additional 100 μL of neural stem cell medium was added after 4 days. Micrographs were acquired 1 week following seeding. To determine the ability to form neurosphere from single cells, we seeded cells of interest at 1 or 5 cells per well in a 96-well plate in triplicate and replenished fresh medium every 2–3 days. Sodium glutamate or *N*-acetyl cysteine was added at 1 mM and refreshed every 2–3 days. Spheres over 50 μm in diameter were counted after 14 days.

### D2-HG analysis

D-2-Hydroxyglutarate Colorimetric Assay Kit (BioVision) was used to measure the intracellular level of D2-HG, as per manufacturer recommendations. Briefly, cell lysates from 1 × 10^5^ cells were split into three parts to determine the absorbance of the sample, 5 nmol D2-HG spiked sample, and sample background. The protein concentration of cell lysate was determined using the BCA Protein Assay Kit (Thermo Scientific). D2-HG was determined in triplicate according to the manufacturer's protocol and expressed as nmol/mg protein.

### Cell proliferation and cell-cycle analysis

Cells expressing luciferase were seeded in a 96-well plate at 100 cells per well in triplicate. Cell proliferation was determined through luciferase activity every 24 hours for 6 consecutive days with a luciferase assay kit (Biotium) or cell viability kit (Promega) and a microplate reader (Turner BioSystems). Relative luciferase units were normalized with background subtraction. Nonlinear regression curve fit was performed using exponential growth equation, and two-way ANOVA was used for the analysis of statistical significance (GraphPad Prism 7.0). Cell-cycle profiling was performed in quadruplicate by labeling the cells with 4′,6-diamidino-2-phenylindole (DAPI) to a final concentration of 10 μg/mL. Cells were then analyzed by flow cytometry (BD FACSCanto, BD Biosciences) with BD FACSDiva Software (BD Biosciences).

### Gene expression

Gene expression analysis at genomic DNA, RNA, and protein levels was performed essentially as described before [15]. Amplicon intensities were quantified using an open-source image analysis platform (Fiji) and normalized to *Actb* expression. Dilutions of primary antibodies for Western blotting are as follows: 1:1000 anti-PDGFB (Santa Cruz Biotechnology), 1:5000 anti-β-actin (Sigma), and 1:500 anti-HA (Abcam).

### Mouse models and bioluminescent imaging

All experiments and procedures involving live mice were approved by the University of Utah Institutional Animal Care and Use Committee. Transplantation of transduced cells into the subcutaneous and intracranial sites and bioluminescent imaging of tumor volume were performed essentially as described previously [58]. Alternatively, subcutaneous tumor growth was measured with an electronic caliper, once a week for 6 weeks. The tumor volume was calculated using the formula (length × width^2^) / 2.

Spontaneous gliomas were generated in *Nes*-*tva* or *Nes*-*tva*;*Cdkn2a* mice as described previously [15, 26]. Briefly, 1–2-day-old newborns were subjected to intracranial injection of DF-1 producer cells expressing genes of interest. The cell number per injection was 3 × 10^4^ PDGFB mixed with equal numbers of IDH1 or IDH1^R132H^ or 3 × 10^4^ PDGFB or mCherry–PDGFB mixed with 1 × 10^5^ YFP* or YFP–IDH1^R132H^. For *Cdkn2a* deletion, additional 1 × 10^5^ DF-1 cells expressing Cre recombinase was included. These mice were terminated by the end of 5 weeks post injection or earlier. The cell number for IDH1–PDGFB or IDH1^R132H^–PDGFB was 5 × 10^5^. For survival analysis, these mice were sacrificed by the end of 8 weeks or earlier if any of the following symptoms was found: severe lethargy, pronounced hydrocephalus, and severe cachexia.

Autopsied brains were embedded in paraffin after formalin fixation, sectioned at 3 μm, and stained with hematoxylin and eosin for histological analysis. For fluorescent microscopy, samples were flash-frozen in liquid nitrogen, embedded in OCT compound, and sectioned at 30 μM thickness with a cryostat microtome (Leica CM1950). Sections were mounted with 30% glycerin containing 10 μg/mL DAPI prior to imaging with a Nikon A1R confocal microscope and NIS-elements confocal software (Nikon Instruments). The image was converted with an open-source image analysis platform (Fiji).

### Immunohistochemistry

Immunohistochemistry was performed in 3-μm sections of formalin-fixed and paraffin-embedded tissues. Primary antibodies and their corresponding dilutions are as follows: 1:200 anti-Ki67 (EMD Millipore), 1:100 anti-PDGFB (Santa Cruz Biotechnology), 1:100 anti-GFP (Novus Biologicals), 1:500 anti-HA (Abcam), 1:200 anti-IDH1^R132H^ (HistoBioTec DIA-H09) or 1:200 anti-IDH1^R132H^ (EMD Millipore), and 1:2000 anti-Olig2 (EMD Millipore). Secondary antibodies used were anti-mouse Fab antibody (Dako) at 1:100 dilution, or ready-to-use kits ImmPRESS™ HRP Anti-Rabbit IgG Polymer Detection Kit (Vector Laboratories), ImmPRESS™ HRP Anti-Goat IgG Polymer Detection Kit (Vector Laboratories), and Mouse on Mouse Elite Peroxidase Kit (Vector Laboratories) followed by DAB (3-3’ diaminobenzidine) as the chromogen and counterstained with hematoxylin.

## SUPPLEMENTARY MATERIALS AND FIGURES



## Abbreviations

D2-HG: D-2-hydroxyglutarate
IDH1: Isocitrate dehydrogenase 1
IDH1^R132H^: IDH1 with histidine substitution at arginine 132
luc: Luciferase
NADPH: Nicotinamide adenine dinucleotide phosphate
PDGFB: Platelet-derived growth factor B
RCAS: Replication-competent avian sarcoma-leukosis virus long-terminal repeat with splice acceptor
YFP: Yellow fluorescent protein
WHO: World Health Organization.
