# Supplementary material for: IDH1R132H is intrinsically tumor-suppressive but functionally attenuated by the glutamate-rich cerebral environment
Source: Oncotarget. 2018 Oct 12;9(80):35100–13. doi: 10.18632/oncotarget.26203 (PMC6205547; doi:10.18632/oncotarget.26203)
Supplement: Supplementary file 1 [file oncotarget-09-35100-s001.pdf]

IDH1^{R132H} is intrinsically tumor-suppressive but functionally attenuated by the glutamate-rich cerebral environment

SUPPLEMENTARY MATERIALS

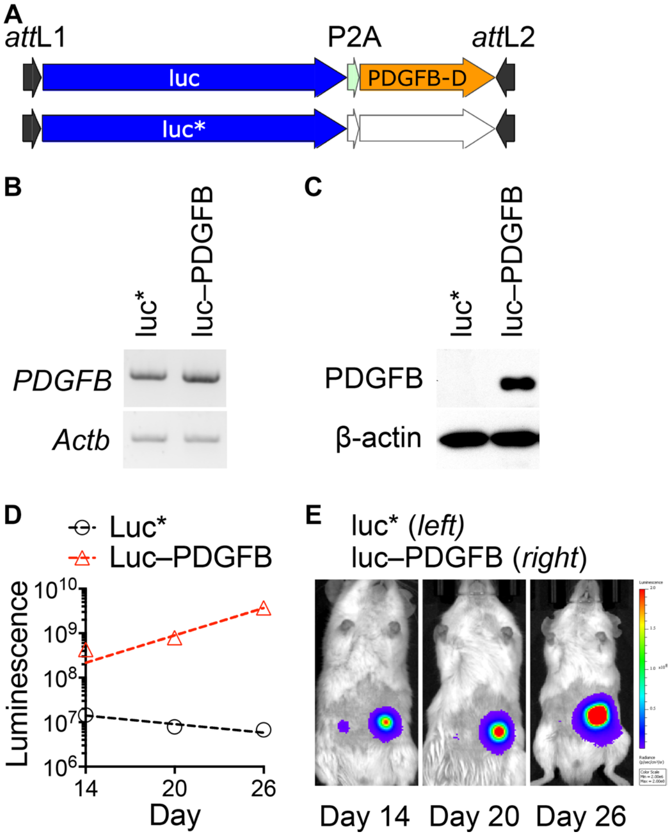

Supplementary Figure 1: Development of luc–PDGFB system for tumor study. (**A**) A schematic drawing of luc–PDGFB and luc* (with an engineered stop codon at P2A) in the context of pDONR221. PDGFB-D denotes PDGFB tagged with DDDDK. P2A and homologous recombination sites *attL1* and *attL2* are indicated. NA1 astrocytes transduced with luc* or luc–PDGFB were analyzed for *PDGFB* expression at mRNA (**B**) and protein (**C**) levels by reverse transcription-PCR and Western blotting, respectively. Analysis of bioluminescent imaging data showing tumor growth derived from subcutaneous injection of 4×10^6 NA1 cells transduced with luc–PDGFB but not with luc* in the NOD/SCID mice. Tumor growth is shown by growth curves (**D**) and images (**E**).

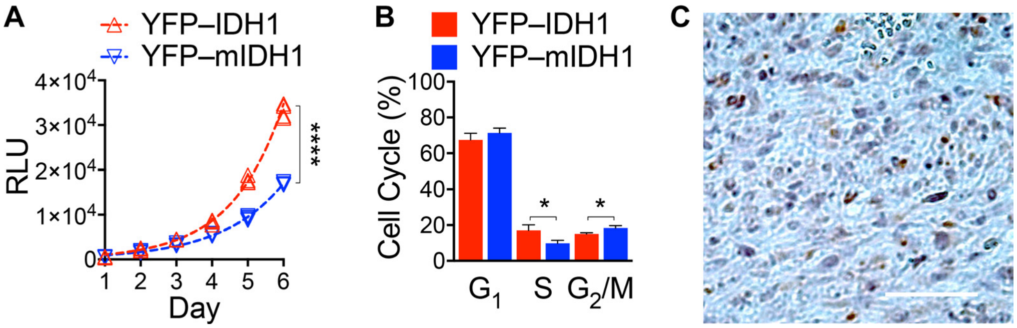

Supplementary Figure 2: Effect of YFP–IDH1 and YFP–IDH1^{R132H} on cell proliferation. NA1 astrocytes transduced with luc–PDGFB showing decreased cell proliferation (**A**, n = 3) and G$_2$/M arrest (**B**, n = 4) upon further transduction with YFP–IDH1^{R132H} (YFP-mIDH1) compared with YFP–IDH1. Nonlinear regression curve fit was performed using exponential growth equation, and two-way ANOVA was used for the analysis of statistical significance. (**C**) Subcutaneous tumor resulting from luc–PDGFB and YFP–IDH1^{R132H} cotransduction showing sparse IDH1^{R132H} staining in the cytoplasm. Unpaired t-tests were performed using two-tailed p values unless insignificant. $^{*}p < 0.05$; $^{****}p < 0.0001$.

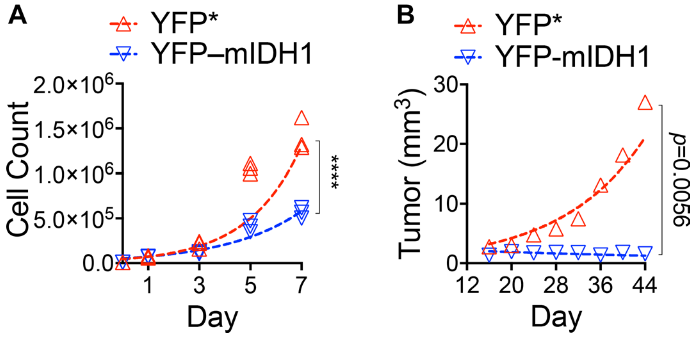

Supplementary Figure 3: IDH1^{R132H} suppression of cell proliferation and subcutaneous tumor growth. (**A**) NA1 astrocytes transduced with mCherry–PDGFB showing significantly decreased cell proliferation (n = 3) after further transduction with YFP–IDH1^{R132H} compared with YFP* (expressing YFP only). $^{****}p < 0.0001$. (**B**) Differential growth curves of subcutaneous tumors (n = 6) derived from these two cell types were plotted with median tumor volumes. Nonlinear regression curve fit was performed using exponential growth equation, and two-way ANOVA was used for the analysis of statistical significance.

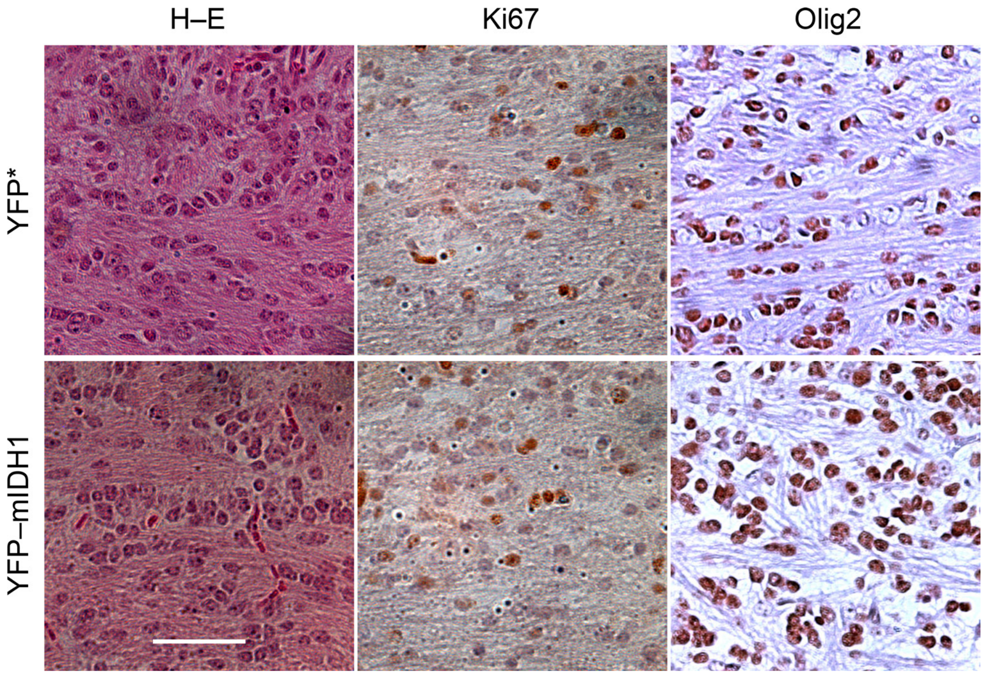

Supplementary Figure 4: Indistinguishable histologies between YFP–IDH1^{R132H} and YFP* gliomas. RCAS/tva mouse models showing similar malignant features including invasion along the white matter tracts, Ki67 and Olig2 staining between PDGFB-induced gliomas cotransduced with YFP–IDH1^{R132H} and YFP*. Scale bar: 50 μm.

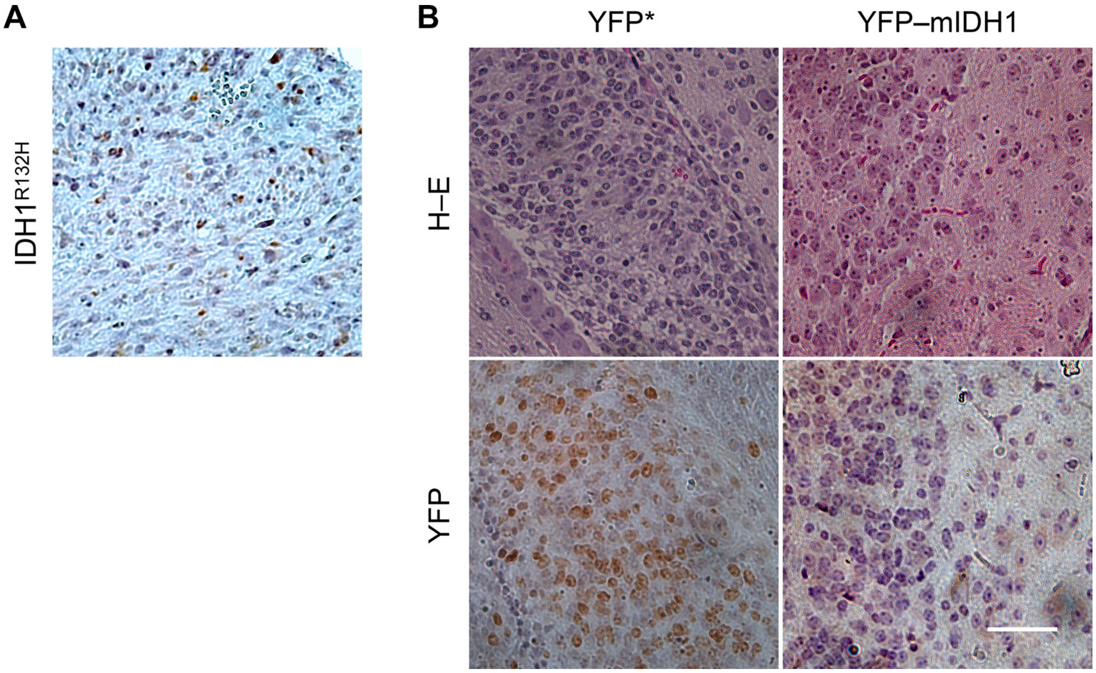

Supplementary Figure 5: Weak IDH1^{R132H} immunohistochemical staining in intracranial tumors. (**A**) Intracranial tumors derived from the transplantation of NA1 co-transduced with luc–PDGFB and YFP–IDH1^{R132H} showing sparse IDH1^{R132H} staining in the cytoplasm. (**B**) RCAS/tva mouse models showing much decreased YFP staining in PDGFB-induced gliomas cotransduced with YFP–IDH1^{R132H} compared with YFP*. Note that the presented images include tumor areas and adjacent brain tissues. Scale bar: 50 μm.

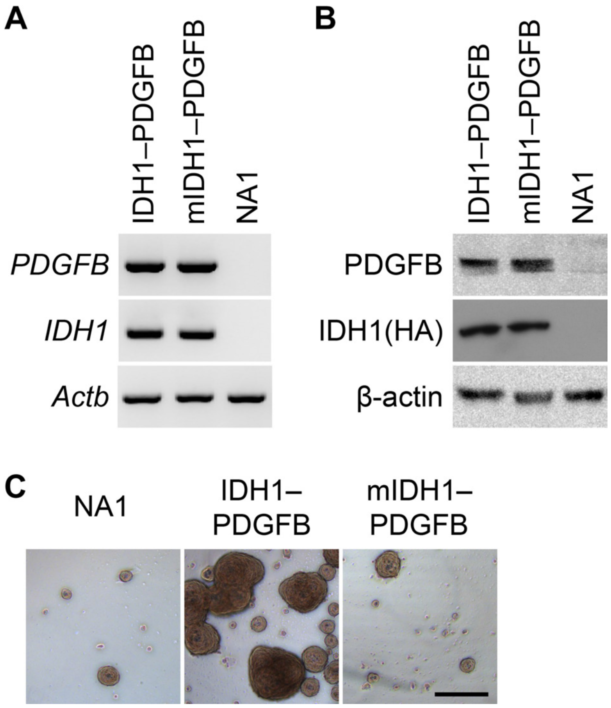

Supplementary Figure 6: Analysis of tandem expression of PDGFB with IDH1 or IDH1^{R132H}. NA1 astrocytes transduced with IDH1–PDGFB and IDH1^{R132H}–PDGFB (mIDH1–PDGFB) were analyzed for equivalent expression of human PDGFB and IDH1 variants at RNA (**A**) and protein (**B**) levels in reference to the endogenous β-actin control. Non-transduced NA1 astrocytes served as negative control. (**C**) IDH1–PDGFB astrocytes showing markedly increased neurosphere growth in comparison with IDH1^{R132H}–PDGFB and NA1 astrocytes. Scale bar: 200 µm.
